# Supplementary material for: Prevalence of sexual dimorphism in mammalian phenotypic traits
Source: Nat Commun. 2017 Jun 26;8:15475. doi: 10.1038/ncomms15475 (PMC5490203; doi:10.1038/ncomms15475)
Supplement: Supplementary Information — Supplementary Figures and Supplementary Tables [file ncomms15475-s1.pdf]

Supplementary Material

Supplementary Figure 1a: Sex as a source of variation: categorical control data.

This figure describes the statistical pipeline developed to assess the role of sex in categorical control data. The role of sex (S) was assessed using a bias reduction logistic regression for each variable of interest (Y). Green nodes indicate the steps taken to manage the multiple testing burden. The first green node represents a potential filter to assess whether the dataset had potential to reach statistical significance at p<0.05. The second green node controls the false discovery rate (FDR) of remaining datasets to 5%.

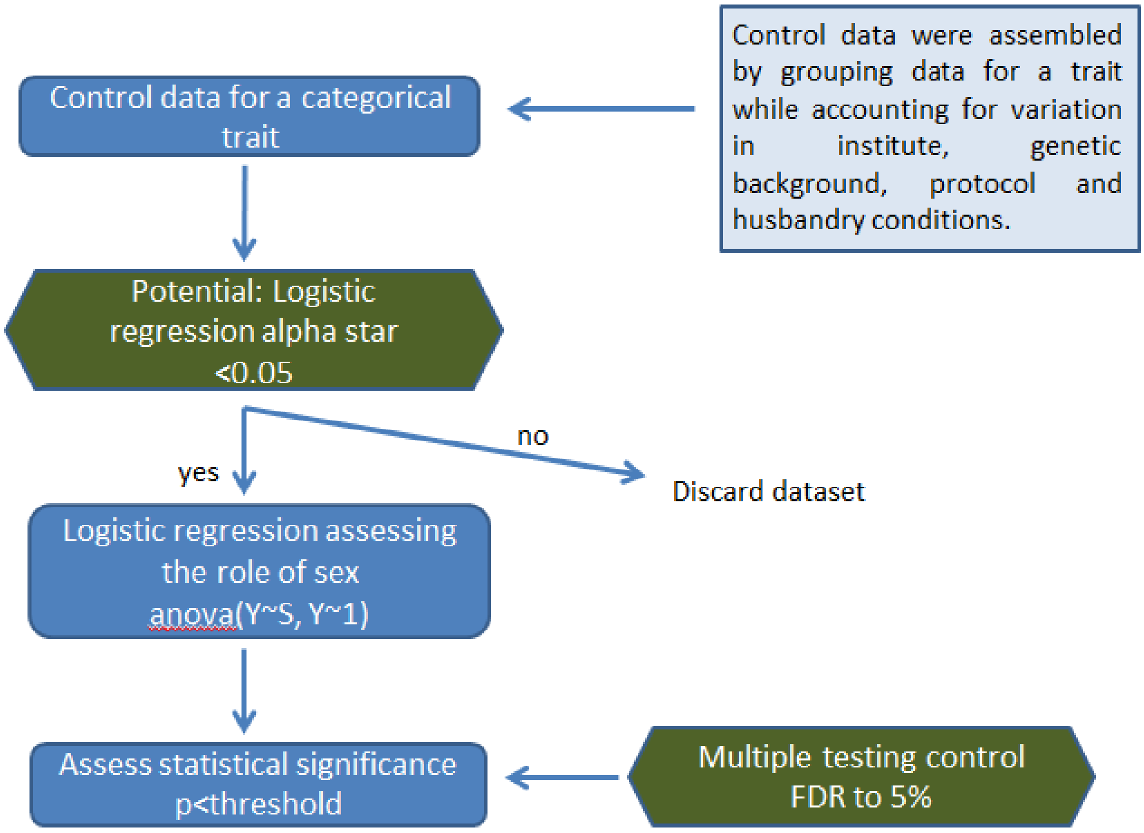

Supplementary Figure 1b: Sex as a source of variation: continuous control data.

This figure describes the statistical pipeline developed to assess the role of sex in continuous control data. The green node indicates the step taken to manage the multiple testing burden where FDR indicates management of the false discovery rate. The data were analysed with a mixed model linear regression for each variable of interest (Y) considering sex (S) and body weight (W) as fixed factors and batch (B) as a random effect. P1 indicates the first pipeline which assessed the role of sex after accounting for body weight differences. P2 represents the second pipeline which assessed the role of sex as an absolute phenotypic difference.

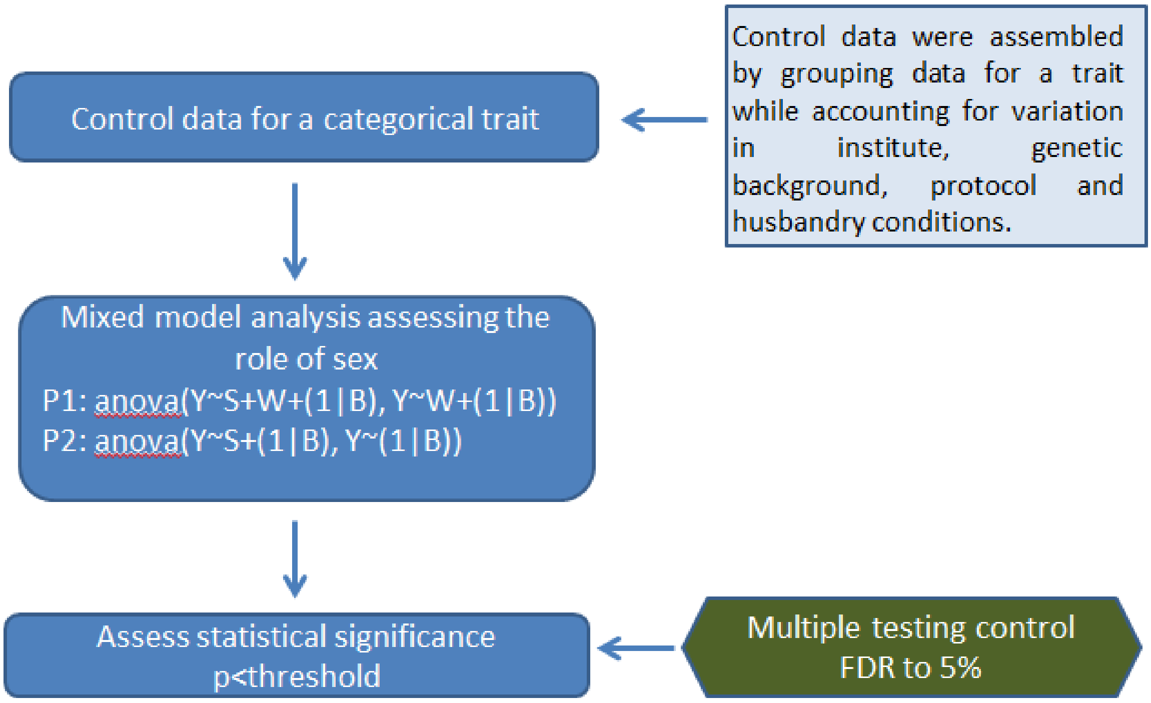

Supplementary Figure 1c: Sex as a modifier of the genotype effect: categorical data.

This figure describes the statistical pipeline developed to assess the role of sex in modifying the genotype effect. Green nodes indicate the steps taken to manage the multiple testing burden where FDR indicates management of the false discovery rate. The data were analysed using a two stage process. Stage 1 used a potential filter (Mantel-Haenszel alpha star) to remove datasets that could not attain statistical significance at p<0.05. The remaining datasets were then assessed with a Mantel-Haenszel mid p test. For stage 2, only datasets that were significant at stage 1 were assessed. Stage 2 also included a potential filter (LR_KO alpha star <0.05) to ensure the dataset had potential to achieve significance prior to testing using a bias reduction logistic regression to compare the abnormality rates between the knockout males and females.

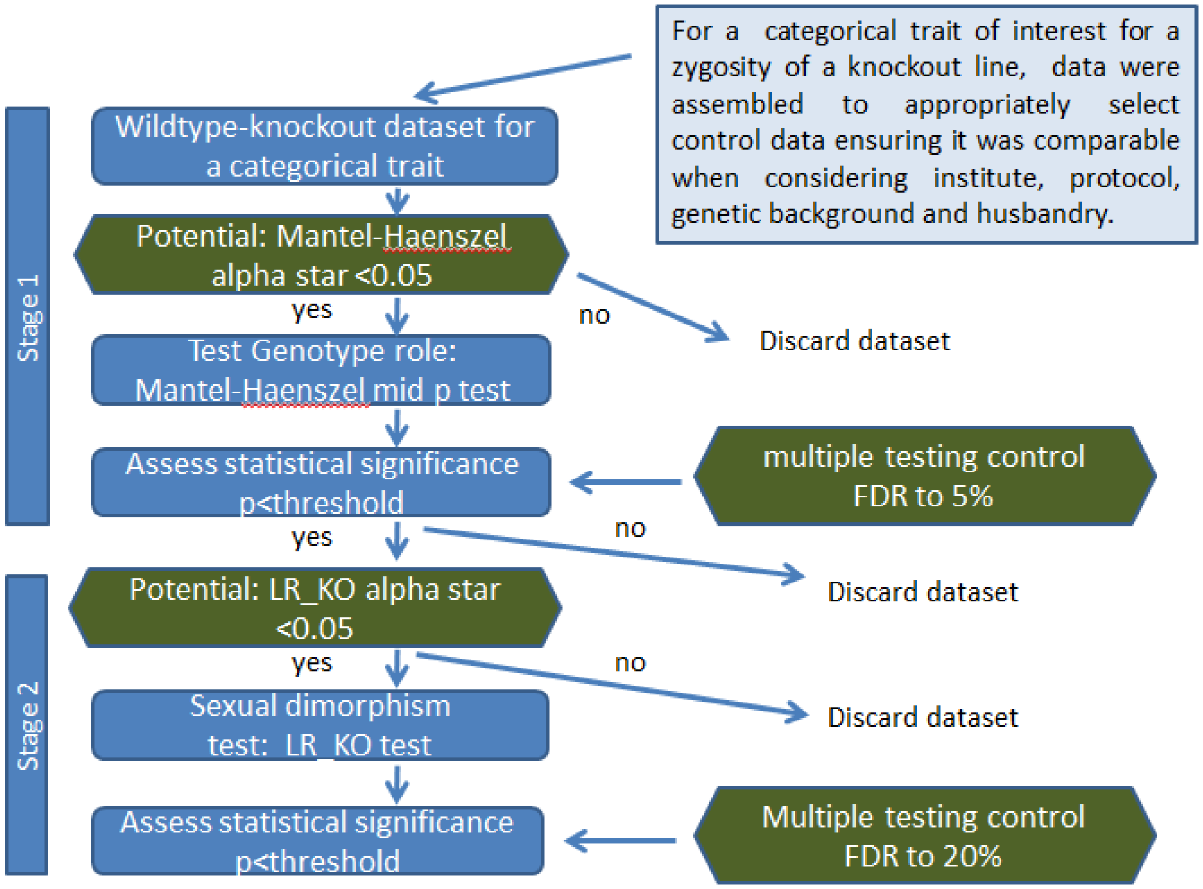

Supplementary Figure 1d: Sex as a modifier of the genotype effect: continuous data.

This figure describes the statistical pipeline developed to assess the role of sex in modifying the genotype effect for continuous traits. Green nodes indicate the steps taken to manage the multiple testing burden where FDR indicates management of the false discovery rate. The data were analysed with a mixed model linear regression for each variable of interest (Y) considering sex (S), genotype (G) and body weight (W) as fixed factors and batch (B) as a random effect. Datasets were first assessed at stage 1 for the role of genotype and then at stage 2 for the role of an interaction between genotype and sex.

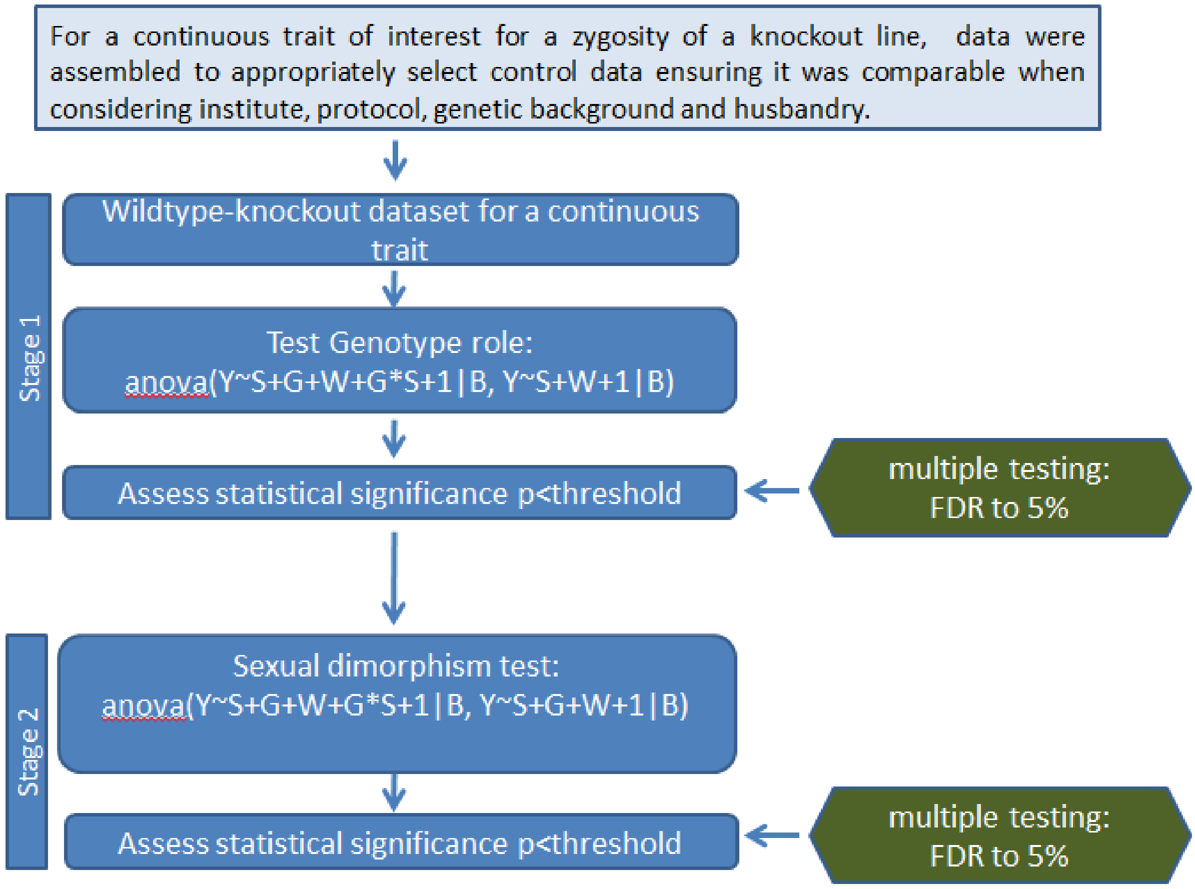

Supplementary Figure 1e: The classification of the genotype effect when studying continuous traits.

The output of the statistical analysis can be used to classify the genotype effect. The classification used the multiple testing adjusted p value from stage 1 testing role of genotype and stage 2 testing for an interaction and the model estimates of the role of genotype by sex using a model threshold of 0.05 to assess significance. For example, if stage 1 was significant but not stage 2, then the classification assigned would be "both sexes equally". Occasionally the procedure implemented will find that there was statistical evidence of sexual dimorphism but when attempting to identify how this occurred and quantifying the effect for each sex, there is insufficient power. In this scenario, the classification returned states that it "cannot classify the effect".

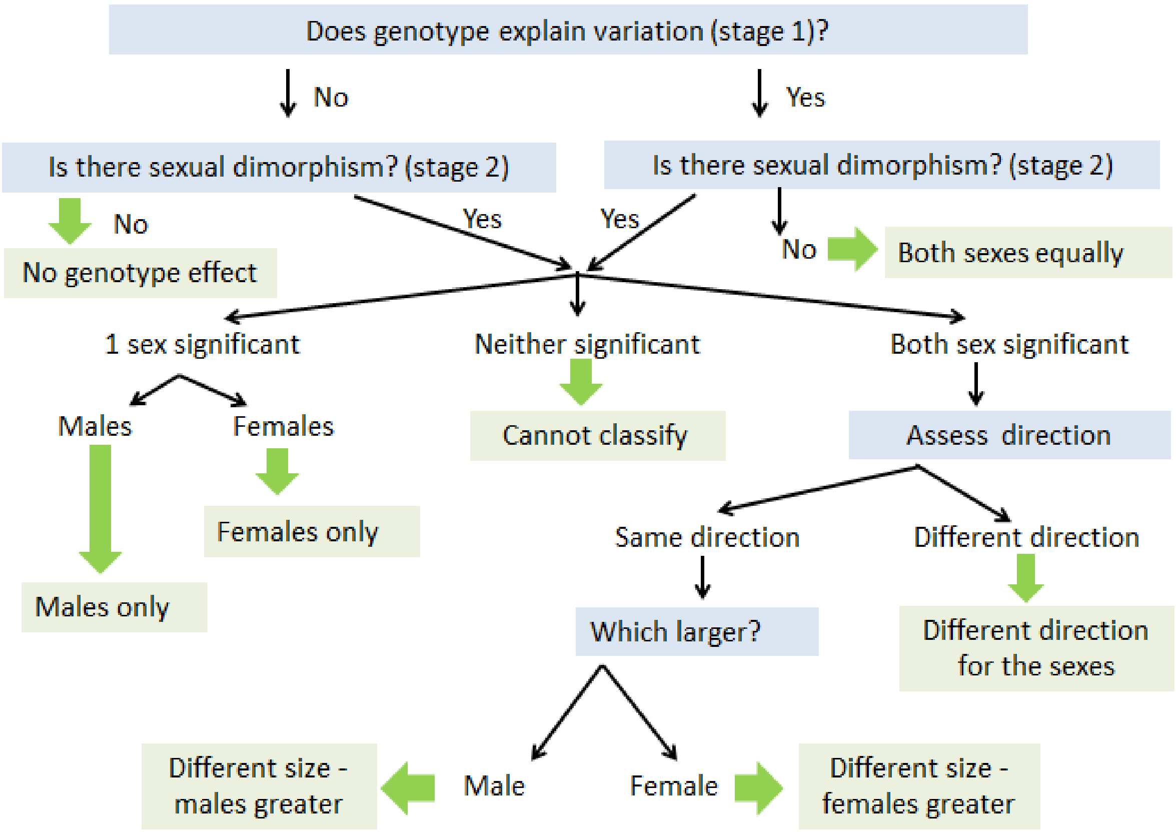

Supplementary Table 1: Sex as a modifier of the genotype effect for categorical data by screen

Screen	Number significant Genotype	Number "female greater"	Number "male Greater"	Genotype effect (%)	Sexual dimorphic (%)
Combined shirpa and dysmorphology	538	46	47	85.26	14.74
Eye morphology	283	25	21	86.01	13.98
X-Ray	237	12	23	91.15	8.85

Supplementary Table 2: Sex as a modifier of the genotype effect for continuous data by screen

Screen	Number datasets significant	Sexual Dimorphic (%)	Genotype Effect (%)
PPI	276	10.51	89.49
DEXA	1912	30.02	69.98
Immunophenotyping	6	16.67	83.33
Calorimetry	30	16.67	83.33
Plasma Chemistry	1714	16.22	83.78
CSD	110	6.36	93.64
ECG	252	10.71	89.29
Echo	79	15.19	84.81
Eye Morphology	92	7.61	92.39
Food efficiency	14	28.57	71.43
Grip Strength	411	16.55	83.45
Haematology	876	15.18	84.82
Organ Weight	133	15.79	84.21
Hole-board Exploration	35	22.86	77.14
Hot Plate	3	0.00	100.00
IPGTT	426	23.24	76.76
Light-Dark Test	173	12.72	87.28
Open Field	1017	7.96	92.04
Rotarod	10	0.00	100.00
Sleep Wake	159	5.66	94.34
Spontaneous breathing	33	6.06	93.94
Tail Suspension	18	5.56	94.44
Urinalysis	2	0.00	100.00
X-Ray	147	12.93	87.07

Supplementary Table 3: Ethical review board information for each phenotyping institute.

ICS Mouse Clinical Institute	Approval Committee: Com'Eth N°17 and French Ministry for Superior Education and Research (MESR)<br>Approval licences: MESR: APAFIS#4789 - 2016040511578546
MRC Harwell	Approval committee: Animal Welfare and Ethical review Board (AWERB)<br>Approval Licence: 30/2890
Nanjing University	Approval committee: IACUC of MARC<br>Approval Licence: NRCMM9
RBRC RIKEN Tsukuba Institute, BioResource Center	Approval committee: The RIKEN Tsukuba Animal Experiments Committee<br>Approval Licence: Exp11-002, 12-002, 13-002, 14-002, 15-002, 16-002<br>Collection, maintenance, storage, breeding and distribution of the mouse resources Exp11-011, 12-011, 13-011, 14-009, 14-017, 15-009, 16-008<br>Phenotying analyses and related studies in mice
The Jackson Laboratory	Approval: The Jackson Laboratory Institutional Animal Care and Use Committee (IACUC)<br>License: NIH Office of Laboratory Animal Welfare (OLAW) assurance # D16-00170<br>Production Grant IACUC Protocol: 14004<br>Phenotyping Grant IACUC Protocol: 11005<br>Phenotyping Grant Supplement IACUC Protocol: 99066<br>Accreditation: AAALACi #000096
The Centre for Phenogenomics	Approval committee: Animal Care Committee (ACC) of The Centre for Phenogenomics (Toronto)<br>Approval Licence: Animal Use Protocol (AUP) 0153, 0275, 0277, 0279
UCD University of California, Davis	Approval committee: UC Davis Institutional Animal Care and Use Committee (IACUC)<br>License: NIH Office of Laboratory Animal Welfare (OLAW) assurance # A3433-01<br>Production and Phenotyping Grants IACUC Protocol: 19075<br>Accreditation: AAALACi #000029 (since 1966)
WTSI Wellcome Trust Sanger Institute	Approval committee: Animal Welfare and Ethical review Board (AWERB)<br>Approval Licence: PPL 80/2076 Valid 27th Nov 2006 - 3rd Jan 2012; PPL 80/2485 valid 3rd Jan 2012 - 3rd Jan 2017

Supplementary Table 4: Number of mice that comprise the $Usp47^{tm1b(EUCOMM)Wtsi}$ (MGI:5605792) dataset presented within Figure 8 of the manuscript.

**Variable**	**Sex**	**Genotype**	**Number mice**
HDL Cholesterol	Male	Wildtype	193
		Knockout	9
	Female	Wildtype	189
		Knockout	7
Bone Mineral content	Male	Wildtype	164
		Knockout	9
	Female	Wildtype	165
		Knockout	7
